# Recent Progress in *N*-Acylethanolamine Research: Biological Functions and Metabolism Regulated by Two Distinct *N*-Acyltransferases: cPLA_2_ε and PLAAT Enzymes

**DOI:** 10.3390/ijms26073359

**Published:** 2025-04-03

**Authors:** Toru Uyama, Sumire Sasaki, Miki Okada-Iwabu, Makoto Murakami

**Affiliations:** 1 Department of Biochemistry, School of Medicine, Kagawa University, 1750-1 Ikenobe, Miki 761-0793, Kagawa, Japan; uyama.toru@kagawa-u.ac.jp (T.U.); sasaki.sumire@kagawa-u.ac.jp (S.S.); iwabu.miki@kagawa-u.ac.jp (M.O.-I.); 2 Laboratory of Microenvironmental and Metabolic Health Science, Center for Disease Biology and Integrative Medicine, Graduate School of Medicine, The University of Tokyo, 7-3-1 Hongo, Bunkyo-ku, Tokyo 113-8655, Japan

**Keywords:** cPLA_2_ε, endocannabinoid, glycerophospholipid, inflammation, *N*-acylethanolamine, *N*-acyltransferase, organelle, PLA_1_, PLA_2_, PLAAT

## Abstract

*N*-Acylethanolamines (NAEs) are a class of lipid mediators that consist of long-chain fatty acids condensed with ethanolamine and exert various biological activities depending on their fatty acyl groups. NAEs are biosynthesized from membrane phospholipids by two-step reactions or alternative multi-step reactions. In the first reaction, *N*-acyltransferases transfer an acyl chain from the *sn*-1 position of phospholipids to the amino group (N position) of phosphatidylethanolamine (PE), generating *N*-acyl-PE (NAPE), a precursor of NAE. So far, two types of *N*-acyltransferases have been identified with different levels of Ca^2+^-dependency: cytosolic phospholipase A_2_ ε (cPLA_2_ε) as a Ca^2+^-dependent *N*-acyltransferase and phospholipase A and acyltransferase (PLAAT) enzymes as Ca^2+^-independent *N*-acyltransferases. Recent in vivo studies using knockout mice with cPLA_2_ε and PLAAT enzymes, combined with lipidomic approaches, have clarified their roles in the skin and brain and in other physiological events. In this review, we summarize the current understanding of the functions and properties of these enzymes.

## 1. Introduction

**Figure 1:**
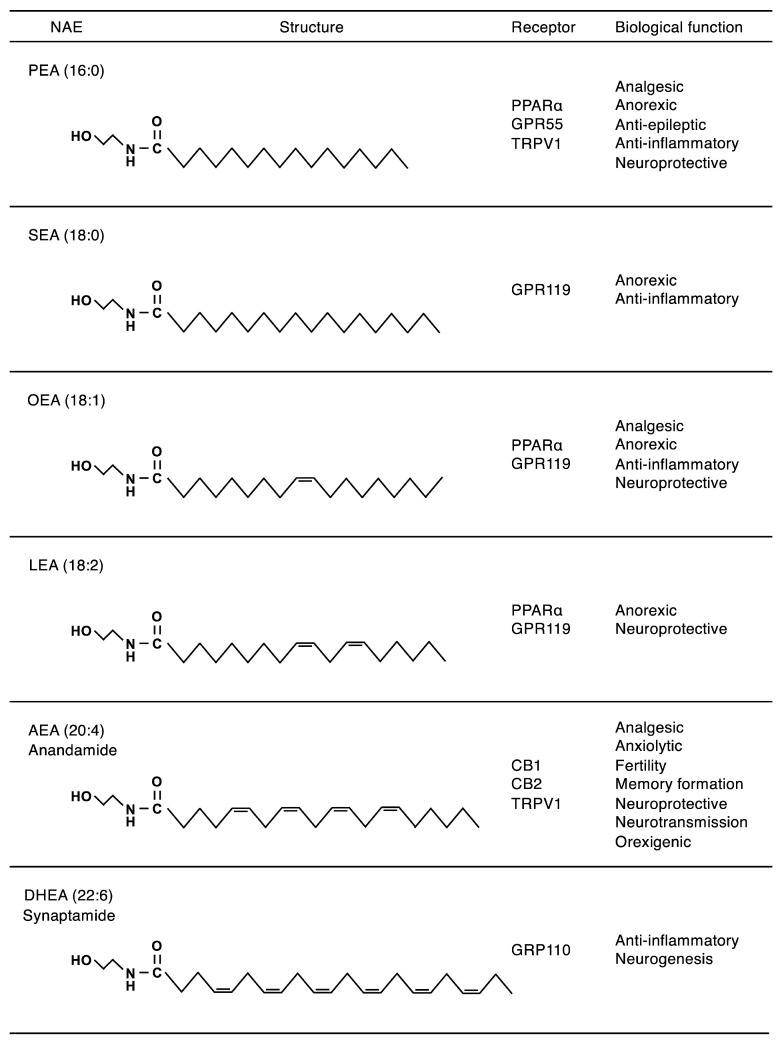
Structures, receptors, and functions of representative NAEs. For more details, please refer to previous reviews [1,2].

*N*-Acylethanolamines (NAEs) are a class of lipid mediators that consist of long-chain fatty acids condensed with ethanolamine and are present in various organisms, including animals and plants (Figure 1) [1,2,3]. NAEs exert various biological activities depending on their fatty acyl groups by acting as ligands on their corresponding receptors. *N*-Arachidonoylethanolamine (AEA/NAE-20:4, also known as anandamide) is an endocannabinoid functioning as an endogenous ligand for the G-protein-coupled cannabinoid receptors CB1 and CB2 and exerts analgesic, anti-inflammatory, anxiolytic, and anti-depressant effects [4,5,6]. *N*-Palmitoylethanolamine (PEA/NAE-16:0) and *N*-oleoylethanolamine (OEA/NAE-18:1) are quantitatively major NAEs in most tissues and exhibit anti-inflammatory and anorexic effects through peroxisome proliferator-activated receptor α (PPARα), a lipid-sensing nuclear receptor, or potentially through other receptors [7,8]. *N*-Docosahexaenoylethanolamine (DHEA/NAE-22:6, also known as synaptamide) plays a role in neurogenesis through GPR110 [9,10].

**Figure 2:**
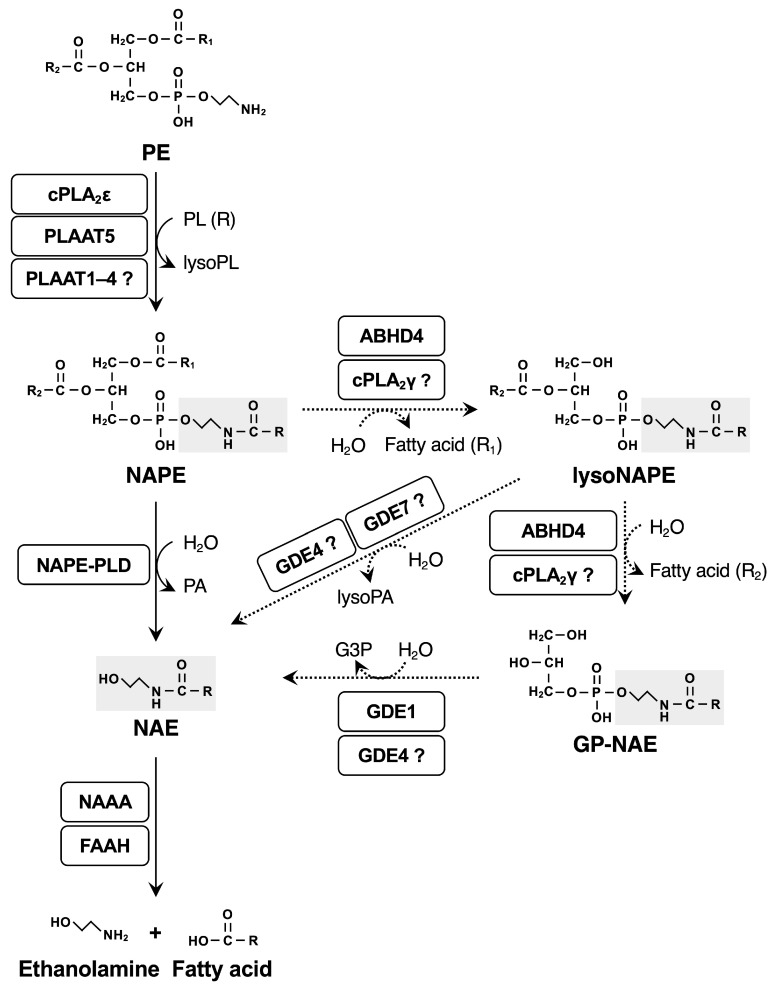
The biosynthetic pathway of NAPE and NAE. NAPE is biosynthesized from PE by cPLA_2_ε or PLAATs and metabolized to NAE directly by NAPE-PLD (a solid arrow) or through alternative pathways (dotted arrows). Closed shades indicate an NAE moiety. The enzymes with a question mark indicate that their enzymatic activities have been detected only in vitro, and their in vivo relevance has not yet been confirmed. G3P, glycerol 3-phosphate; PL, phospholipid; NAAA, *N*-acylethanolamine-hydrolyzing acid amidase; FAAH, fatty acid amid hydrolase.

NAEs are biosynthesized from membrane phospholipids by two-step reactions or alternative multi-step reactions (Figure 2). In the first reaction, *N*-acyltransferases transfer an acyl chain from the *sn*-1 position of phospholipids to the amino group (N position) of phosphatidylethanolamine (PE), generating *N*-acyl-PE (NAPE), a precursor of NAE. In this context, *N*-acyltransferases are different from CoA-dependent acyltransferases and are regarded as transacylases. NAPE, a unique phospholipid with three acyl chains, is expected to be embedded in the membrane bilayer, providing a membrane-stabilizing property [11]. The resultant NAPE is converted to NAE directly by NAPE-hydrolyzing phospholipase D (NAPE-PLD) or through alternative pathways consisting of multi-step reactions, which may include α/β-hydrolase domain-containing 4 (ABHD4), cytosolic phospholipase A_2_ γ (cPLA_2_γ), and glycerophosphodiesterases (GDEs) such as GDE1, GDE4, and GDE7. So far, two types of *N*-acyltransferases have been identified with different Ca^2+^-dependency: cytosolic phospholipase A_2_ ε (cPLA_2_ε) as a Ca^2+^-dependent *N*-acyltransferase [12] and the phospholipase A and acyltransferase (PLAAT) family of enzymes comprising five members (PLAAT1–5) as Ca^2+^-independent *N*-acyltransferases [13]. Recent in vivo studies using knockout mice with cPLA_2_ε and PLAAT enzymes, combined with lipidomic approaches, have clarified their roles in the skin and brain as well as in other physiological events. In this review, we summarize the current understanding of the functions and properties of these enzymes.

## 2. cPLA_2_ε

### 2.1. cPLA_2_ε Functions as a Ca^2+^-Dependent N-Acyltransferase

**Figure 3:**
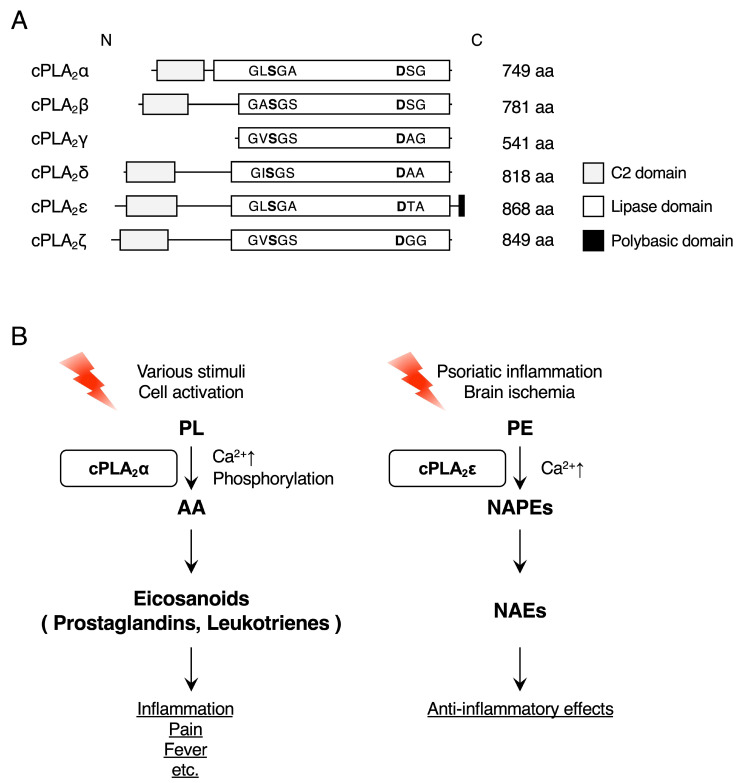
Structures of cPLA_2_ proteins and the enzymatic properties of cPLA_2_α and cPLA_2_ε. (**A**) Structures of human cPLA_2_ proteins. The Ser/Asp catalytic dyad in the lipase domain of each cPLA_2_ isoform is shown in bold. The N-terminal C2 domain, which mediates Ca^2+^-dependent membrane translocation, is characteristic of the cPLA_2_ family (except for cPLA_2_g). (**B**) Upon cell activation in response to various stimuli, cPLA_2_α is activated by an increase in cytosolic Ca^2+^ concentration and phosphorylation to selectively release arachidonic acid through its PLA_2_ activity. The cPLA_2_α-driven arachidonic acid is converted to eicosanoids (prostaglandins and leukotrienes) to exert various biological effects, such as inflammation, pain, and fever, among others. In the models of psoriatic inflammation and brain ischemia, cPLA_2_ε activated by Ca^2+^ (or possibly other unknown mechanisms) produces NAPEs and NAEs, which exert anti-inflammatory effects. The arrows indicate the flow of the metabolic pathways. AA, arachidonic acid; PL, phospholipid.

Ca^2+^-dependent *N*-acyltransferase activity was first detected in dog hearts [14] and brains [15], and its properties were characterized using enzyme sources partially purified from rat tissues [16,17,18]. The enzyme was membrane-bound and selectively utilized an acyl chain from the *sn*-1 position of phospholipids as an acyl donor, generating NAPEs (Figure 2). The activity was significantly enhanced by the cysteine-reducing reagent dithiothreitol and was inhibited by chemicals with alkylate serine (phenylmethylsulfonyl fluoride), cysteine (dithionitrobenzoic acid), and histidine (*p*-bromophenacylbromide) residues [17]. Ca^2+^ in the order of millimolar concentrations was required for the full activity, and consistently, the treatment of rat cortical neurons with the Ca^2+^ ionophore ionomycin markedly increased NAPE generation [17,18]. By using activity-based protein profiling on the mouse brain, Cravatt and colleagues identified cPLA_2_ε, also known as PLA2G4E or group IVE PLA_2_, as a Ca^2+^-dependent *N*-acyltransferase (Figure 3) [12]. As observed with the partially purified enzyme from several sources, recombinant cPLA_2_ε directly transfers the *sn*-1, rather than *sn*-2, fatty acyl chain of donor phospholipids, such as phosphatidylcholine (PC), to the N position of the acceptor PE [12]. When overexpressed in several mammalian cells, cPLA_2_ε generates large amounts of NAPEs and their downstream metabolites, including NAEs and glycerophospho-NAEs (GP-NAEs), which undergo further enhancement by ionomycin treatment (Figure 2) [12,19,20]. In particular, there are marked increases in NAPEs and NAEs with a saturated or monounsaturated acyl chain (e.g., 16:0, 18:0, or 18:1) at the N position. These profiles of NAPE and NAE species are consistent with the above-mentioned specificity of cPLA_2_ε for the *sn*-1 position of phospholipids because saturated and monounsaturated rather than polyunsaturated acyl chains are mainly bound to the *sn*-1 position.

cPLA_2_ε/PLA2G4E belongs to the cytosolic phospholipase A_2_ (cPLA_2_) family (Figure 3A), which is also known as group IV PLA_2_ and is composed of six isoforms (α—ζ or IVA—IVF) [21]. These enzymes have molecular weights ranging from 60 to 100 kDa and have a Ser-Asp catalytic dyad in the lipase domain. Except for cPLA_2_γ (see below), they have a C2 domain, which binds to Ca^2+^ and regulates membrane association [22] in the N-terminal region. The enzymatic properties of these cPLA_2_ isoforms are summarized in Table 1. It is well established that cPLA_2_a/PLA2G4A, the prototypic member of this family, plays a crucial role in the generation of eicosanoids (prostaglandins and leukotrienes) by selectively releasing arachidonic acid from the *sn*-2 position of phospholipids (i.e., genuine PLA_2_ reaction) in various cell types (Figure 3B, left) [23]. In contrast, when overexpressed in HEK293 cells or keratinocytes, cPLA_2_ε, but not other cPLA_2_ isoforms, generates NAPEs and NAEs in a Ca^2+^-dependent manner (Figure 3B, right) [24,25]. Anionic phospholipids, including phosphatidylserine (PS), phosphatidic acid (PA), and phosphatidylinositol 4,5-bisphosphate (PIP_2_) and enhance the enzyme activity of cPLA_2_ε in correlation with its membrane binding [20,26]. cPLA_2_ε localizes to PS-rich organelles, such as lysosomes and early endosomes, and a reduction in PS levels by blocking PS biosynthesis concomitantly decreases the protein levels of membrane-bound cPLA_2_ε [20,26]. cPLA_2_ε has also been reported to be targeted to the membrane compartments rich in phosphoinositides, especially PIP_2_, through a C-terminal stretch of positively charged amino acids and to regulate trafficking processes within the clathrin-independent endocytic and recycling routes [27]. Since a catalytically inactive mutant of cPLA_2_ε fails to affect the trafficking processes, lipids produced by cPLA_2_ε, possibly NAPEs and NAEs, may be crucial for this event.

The overall trend for the magnitude order of NAPE and NAE levels in normal tissues of adult mice is skin > muscle > stomach > brain > heart > liver > kidney, which is roughly even if not solely correlated with that of cPLA_2_ε expression (muscle > stomach > skin > brain > heart > liver > kidney) [28]. In these tissues, major NAE species are NAE-16:0, -18:0, and -18:1, which is consistent with the fact that the *sn*-1 fatty acid (mostly saturated or monounsaturated) of PC is preferentially utilized for transacylation by cPLA_2_ε to give rise to NAPEs and thereby NAEs.

### 2.2. cPLA_2_ε in the Brain

#### 2.2.1. Neonatal Development

The brain is one of the organs in which NAPEs and NAEs are abundant [29], and Ca^2+^-dependent *N*-acyltransferase activity is high, especially in neonatal mouse brains [16,17,30,31]. cPLA_2_ε expression in mouse brains is age-dependent and begins to increase on embryonic day 17 (E17), reaching the highest expression between postnatal day 0 (P0) and P7 and declining by P28 [29]. Consistent with this expression pattern, Ca^2+^-dependent *N*-acyltransferase activity is detectable between E17 and P28, with the highest activity at P7. Cravatt and colleagues have also shown that the expression and activity of cPLA_2_ε are age-dependent and approximately four-fold higher in the brain at P1 than at 10 weeks of age [12]. Importantly, Ca^2+^-dependent *N*-acyltransferase activity is abolished in the brain of cPLA_2_ε-deficient (*Pla2g4e*^−/−^) mice, confirming that cPLA_2_ε functions as a major Ca^2+^-dependent *N*-acyltransferase in the brain, especially at younger ages [29]. Lipidomic analysis of the brain from wild-type (WT) mice reveals an age-dependent increase in NAPE levels, while cPLA_2_ε expression is the highest at P7 and decreases thereafter. NAPE levels in the brain from *Pla2g4e*^−/−^ mice at P7 are decreased by half, while those at P30 are unchanged compared to WT mice [29]. These results suggest that cPLA_2_ε partially contributes to NAPE generation in the brain only at early ages but not thereafter and that the brain expresses *N*-acyltransferase(s) other than cPLA_2_ε.

#### 2.2.2. Ischemia

Although NAPEs and NAEs are known to accumulate markedly under certain pathological conditions, such as brain ischemia, the enzyme responsible for their production has not yet been identified [30,31,32,33,34,35]. By using an ex vivo model of brain ischemia, we clarified that cPLA_2_ε is the *N*-acyltransferase that robustly produces NAPEs and NAEs in the ischemic brain (Figure 3B) [29]. In this model, NAPEs are increased more than 10-fold over their basal level in the WT brain. As for the molecular species, remarkable increases are observed for NAPEs possessing a saturated or monounsaturated acyl chain at the N position. Similarly, NAEs are increased more than 200-fold with a preference for saturated and monounsaturated NAEs, including PEA/NAE-16:0, *N*-stearoylethanolamine (SEA/NAE-18:0), and OEA/NAE-18:1. The molecular species of NAPEs and NAEs that increased in the brain ischemia model are consistent with the regiospecificity of the recombinant cPLA_2_ε protein, which prefers the *sn*-1 position of phospholipids as an acyl donor [12]. These increases in NAPEs and NAEs are not detectable in the brain of *Pla2g4e*^−/−^ mice, indicating that cPLA_2_ε is indeed responsible for the generation of NAPEs and NAEs in the ischemic brain.

NAEs that are increased in the ischemic brain may exert cytoprotective effects on injured cells and tissues. The administration of anti-inflammatory NAEs such as PEA/NAE-16:0 or OEA/NAE-18:1 (Figure 1) has been shown to reduce infarct volume in the brain in cerebral ischemia/reperfusion models through PPARα signaling [36,37]. In Alzheimer’s disease (AD), cPLA_2_ε expression is significantly decreased in the brain of late-stage patients but not in early-stage patients, suggesting that cPLA_2_ε may have protective effects on AD progression and/or the onset of dementia, possibly through the generation of NAEs [38]. Indeed, the adenoviral delivery of cPLA_2_ε in hippocampal neurons ameliorated cognitive defects in a mouse AD model [38]. Whole-exome sequencing has identified *PLA2G4E* as a risk gene for panic disorder, a neuropsychiatric disease characterized by recurrent and unexpected panic attacks, subsequent anticipatory anxiety, and phobic avoidance [39]. Further analysis of *Pla2g4e*^−/−^ mice will provide insights into the physiological and pathophysiological significance of NAPEs and NAEs in brain diseases. Besides the cytoprotective effects of NAPEs and NAEs, the fluctuating properties of these lipids may be available as useful biomarkers for brain injury.

### 2.3. cPLA_2_ε in the Skin

In both mouse and human skins, cPLA_2_ε is expressed in epidermal keratinocytes and is highly elevated in psoriatic skins [25]. Consistently, cPLA_2_ε expression is upregulated by psoriasis-related cytokines such as IL-17A and TNFα in cultured keratinocytes [40]. In the imiquimod (IMQ)-induced mouse psoriasis model, the increased expression of cPLA_2_ε is accompanied by the concomitant production of NAE-related lipids, including NAPEs, GP-NAEs, and NAEs [25]. *Pla2g4e*^−/−^ mice show exacerbated IMQ-induced psoriatic inflammation, with a striking reduction in NAE-related lipids in IMQ-treated and even normal skin [25]. In contrast, neither arachidonic acid-derived eicosanoids, eicosapentaenoic acid (EPA)- docosahexaenoic acid (DHA)-derived oxylipins, nor lysophospholipid-derived mediators are decreased in *Pla2g4e*^−/−^ skin, implying the specific role of cPLA_2_ε in the NAE-biosynthetic pathway. Moreover, treatment with exogenous NAE-16:0, -18:1, and -20:0 alleviates psoriatic responses in IMQ-challenged skin in vivo and cytokine-stimulated keratinocytes in vitro through a pathway partially dependent on PPARα. Thus, cPLA_2_ε induced in keratinocytes by psoriatic cytokines is responsible for the generation of NAE-related lipids, which constrain psoriatic inflammation as a negative feedback mechanism. These findings are compatible with a study demonstrating that an increase in NAEs by the pharmacological inhibition of NAE amidase, which splits NAE into fatty acid and ethanolamine, ameliorates allergic dermatitis in mice [41]. In contrast, pro-inflammatory eicosanoids such as TXA_2_ and LTB_4_, which are likely produced by cPLA_2_α, contribute to the exacerbation of psoriasis [42,43], highlighting distinct roles of the two cPLA_2_ isoforms in the same skin disease by mobilizing distinct classes of lipid mediators.

### 2.4. cPLA_2_ε in Other Tissues

As observed in the ischemic brain and psoriatic skin, the levels of NAEs are markedly reduced in the skeletal muscle and stomach of *Pla2g4e*^−/−^ mice [28]. Since PEA/NAE-16:0 and OEA/NAE-18:1 act as ligands for PPARα, which improves mitochondrial function and energy expenditure, cPLA_2_ε may contribute to the regulation of energy homeostasis by generating these NAE species in skeletal muscle. In patients with chronic muscle pain, the increase in pain intensity following low-force exercise is associated with low levels of PEA/NAE-16:0 and SEA/NAE-18:0 [7,44], suggesting that cPLA_2_ε-driven NAEs may have an anti-nociceptive role in the muscle. In the gut, OEA/NAE-18:1 exerts an anorexic effect by regulating food intake and systemic lipid metabolism via acting on PPARα, GPR119, and TRPV1 receptors [8,45]. The gut-specific ablation of NAPE-PLD, an enzyme that catalyzes the conversion of NAPEs to NAEs, leads to hyperphasia, reduced energy expenditure, hepatic steatosis, and dysbiosis via the gut-to-brain axis [46,47]. Thus, the cPLA_2_ε-NAE pathway in the gut may contribute to the control of appetite and systemic metabolism.

Although NAEs are detectable in the liver, kidney, and heart at lower levels, they are not significantly decreased by cPLA_2_ε deficiency [28], suggesting that NAE biosynthesis in these tissues depends largely on enzymes other than cPLA_2_ε. However, the possibility that cPLA_2_e would be induced or activated in these tissues and participate in the synthesis of NAE-related lipids under certain pathophysiological conditions cannot be ruled out. During fasting, histamine released from extra-hepatic mast cells enters the liver through the portal circulation, acts on the histamine H_1_ receptor, and promotes the synthesis of OEA/NAE-18:1, which then activates PPARα toward increased ketogenesis [48]. In this situation, H_1_-evoked Ca^2+^ signaling might trigger cPLA_2_ε activation for OEA/NAE-18:1 production in the liver, although this point has not been firmly confirmed.

### 2.5. cPLA_2_γ and ABHD4 Potentially Contribute to the Conversion of NAPEs to NAEs

Unlike other cPLA_2_ isoforms, cPLA_2_γ/PLA2G4C (group IVC PLA_2_) lacks the C2 domain (Figure 3A), and its enzyme activity is not regulated by Ca^2+^. cPLA_2_γ possesses multiple enzyme activities, including PLA_2_, lysophospholipase, and transacylase (*O*-acyltransferase) activities (Table 1), and has been implicated in phospholipid remodeling [49]. The purified recombinant cPLA_2_γ sequentially hydrolyzes both the *sn*-1 and *sn*-2 acyl chains of NAPEs by its PLA_2_ and lysophospholipase activities, generating lysoNAPEs and then GP-NAEs (Figure 2) [24]. Consistent with these activities, the transfection of cPLA_2_γ in HEK293 cells overexpressing cPLA_2_ε, which produces large quantities of NAPEs, increases lysoNAPEs and NAEs, presumably via GP-NAEs. However, it remains to be elucidated whether cPLA_2_γ could participate in NAE generation in vivo.

ABHD4, a member of the ABHD family that contains at least 23 isoforms with various lipid hydrolase activities [50,51], also catalyzes the same reactions as cPLA_2_γ in terms of NAE synthesis [52]. Lipidomic analysis of the brain from ABHD4-deficient (*Abhd4*^−/−^) mice demonstrates partial reductions in GP-NAEs and plasmalogen-based lysoNAPEs [53]. In addition, a decrease in *N*-acyl-lysoPSs (lysoNAPSs), another type of *N*-acylated phospholipid, is evident in the *Abhd4*^−/−^ brain. Enzymatic examination reveals that ABHD4 is indeed capable of hydrolyzing *N*-acyl-PSs (NAPSs) to lysoNAPSs. NAPSs are potential precursors of *N*-acylserines, which are implicated in inflammation and neuroprotection [54,55], and further studies on ABHD4 may provide insight into the biosynthetic regulations and physiological roles of this unique class of lipids.

## 3. The PLAAT Family

### 3.1. The PLAAT Family Functions as Ca^2+^-Independent N-/O-Acyltransferases and PLA_1_/A_2_s

**Figure 4:**
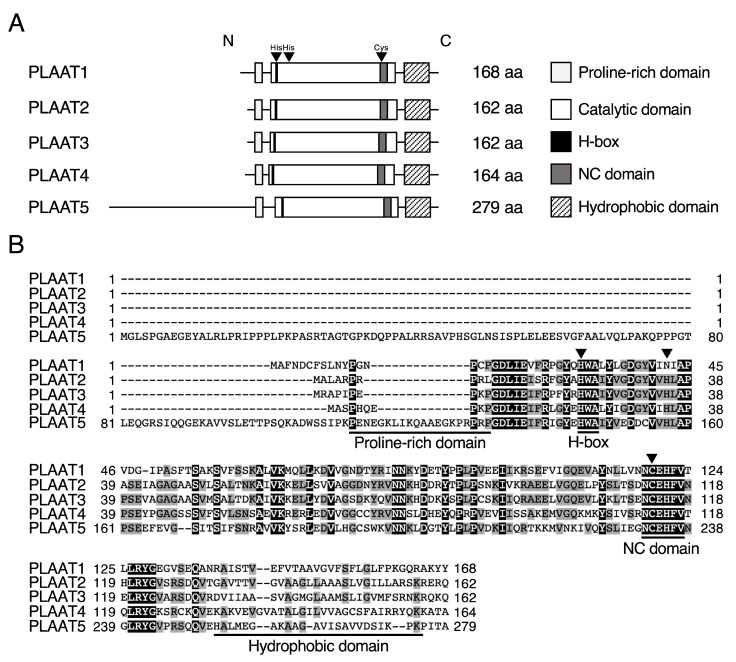
Structures of PLAAT proteins. (**A**) Structures of human PLAAT proteins. (**B**) The amino acid sequences of human PLAAT1−5 are aligned using the program GENETYX-MAC (version 20). Closed and shaded boxes indicate conserved amino acids in all five or more than three proteins, respectively. The arrowheads show the residues forming the catalytic triad.

The PLAAT family comprises five members (PLAAT1–5) in humans and three members (PLAAT1, 3, and 5) in rodents, all of which function as Ca^2+^-independent *N*- or *O*-acyltransferases (transacylases) and/or PLA_1_/A_2_s (Figure 4). PLAAT3 is often referred to as PLA2G16 (group XVI PLA_2_) along with the classical PLA_2_ nomenclature or adipose-specific PLA_2_ (AdPLA_2_) since it is highly induced during adipocyte differentiation [56]. They are conserved in vertebrates, including zebrafish [57], mice [58,59,60], and humans [58]. These proteins are small in size (162–279 amino acids in humans) and share similar domain structures with each other, consisting of a proline-rich domain, a catalytic domain containing an H-box and NC domain, and a hydrophobic domain (Figure 4A). A catalytically important Cys and two His residues, which are present in the catalytic domain, form a Cys-His-His catalytic triad [61,62] (Figure 4B). PLAAT1 has an Asn residue instead of a second His residue and forms a Cys-His-Asn catalytic triad. The substitution of any of these amino acids in PLAAT3 with Ala or Ser abolishes its enzyme activity [60,62]. Similar findings were also observed with the point mutants of PLAAT1, 2, and 5 [13,59,63].

**Figure 5:**
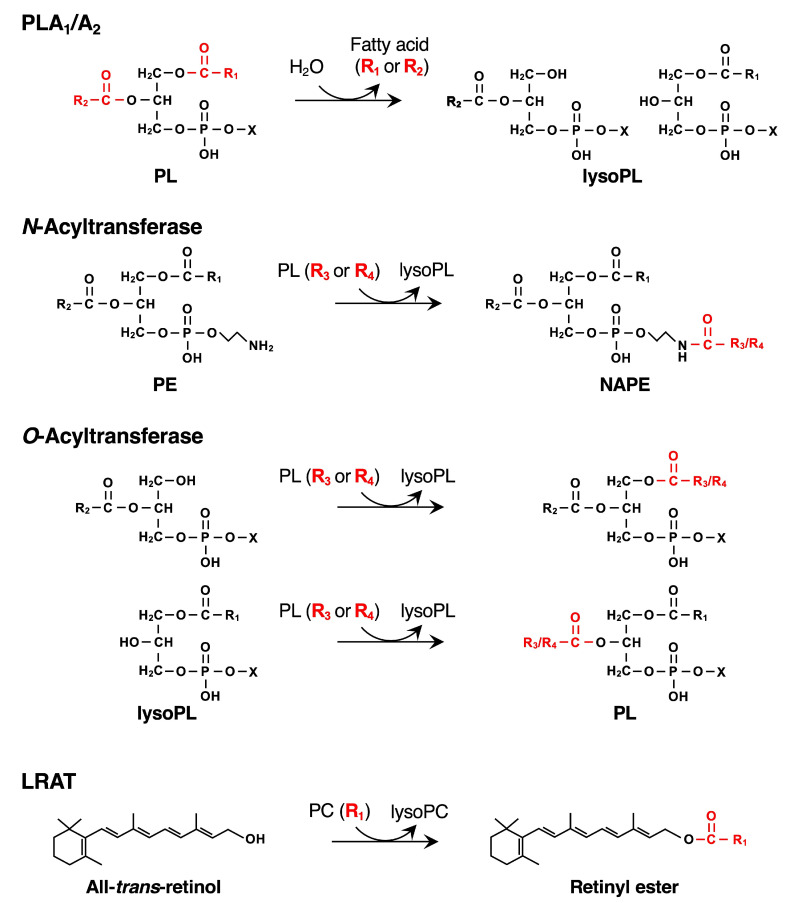
Enzymatic reactions catalyzed by PLAATs and LRAT. The acyl moieties hydrolyzed or transferred by PLAATs and LPAT are shown in red. lysoPL, lysophospholipid.

We and other groups have shown that all five PLAAT proteins catalyze PLA_1_/A_2_ and/or CoA-independent acyltransferase (also regarded as transacylase) reactions in vitro, namely PLA_1_/A_2_ activity, releasing a free fatty acid by hydrolyzing an ester bond at the *sn*-1 or -2 position of phospholipid, *N*-acyltransferase activity producing NAPE, and *O*-acyltransferase activity by transferring an acyl chain from the phospholipid directly to the hydroxyl group of lysophospholipid without the requirement of a CoA-dependent reaction (Figure 5) [16,58,59,60,64]. PLAAT1 and 5 show relatively higher acyltransferase activities, PLAAT3 and 4 prefer PLA_1_/A_2_ activity, and PLAAT2 comparably exhibits both activities (Table 2). Consistently, the overexpression of PLAAT1 and 2 in COS7 cells generate large quantities of NAPEs and NAEs; PLAAT4 and 5 produce them to some extent; and PLAAT3 does not [13]. The siRNA-mediated silencing of PLAAT1 in ATDC5 cells or PLAAT2 in HeLa cells decreases endogenous NAPE levels, suggesting that the expression levels of these PLAAT proteins correlate, at least in part, with NAPE synthesis at the cellular level [13,63]. PLAAT proteins also show structural similarity to lecithin-retinol acyltransferase (LRAT), a key enzyme in vitamin A metabolism [65,66]. LRAT produces retinyl ester, a storage form of vitamin A, by transferring an acyl chain from the *sn*-1 position of PC to all-*trans*-retinol (Figure 5) [67,68,69]. Although this reaction type of LRAT is analogous to that of *N*-acyltransferase in terms of using an acyl chain of phospholipids as an acyl donor, LRAT does not exhibit *N*-acyltransferase activity, and conversely, PLAATs do not exhibit LRAT activity [16].

### 3.2. PLAAT5 Functions as a Ca^2+^-Independent N-Acyltransferase Producing Anti-Inflammatory NAEs in the Testis

PLAAT5 is specifically expressed in the testis, which contains NAPEs and NAEs abundantly [70] and exhibits the highest Ca^2+^-independent *N*-acyltransferase activity among various mouse tissues [16,59]. By using PLAAT5-deficient (*Plaat5*^−/−^) mice, we obtained evidence that PLAAT5 is a testicular Ca^2+^-independent *N*-acyltransferase that produces anti-inflammatory NAEs [71]. *Plaat5*^−/−^ mice are born at Mendelian ratios and are apparently healthy. Ca^2+^-independent *N*-acyltransferase activity is hardly detected in the testis of *Plaat5*^−/−^ mice, indicating that PLAAT5 is a major Ca^2+^-independent *N*-acyltransferase in the testis. Although PLAAT1 is also expressed in the testis at a substantial level, *N*-acyltransferase activity in the testes does not significantly differ between PLAAT1-deficient (*Plaat1*^−/−^) and WT mice.

Lipidomic analysis of the testis reveals that NAE levels are significantly decreased in *Plaat5*^−/−^ mice, with PEA/NAE-16:0, the most abundant NAE in this tissue, being decreased by 64% [71]. In particular, PLAAT5 deficiency has a greater influence on polyunsaturated NAEs, with the levels of AEA/NAE-20:4, *N*-docosapentaenoylethanolamine (DPEA/NAE-22:5), and DHEA/NAE-22:6 being decreased by 87%, 85%, and 82%, respectively. Since polyunsaturated fatty acids are mainly bound to the *sn*-2 position of phospholipids, PLAAT5 seems to preferentially transfer an acyl chain donor from the *sn*-2 position to give rise to NAPE. This regiospecificity is consistent with the enzymatic properties of the purified recombinant PLAAT5 protein, which prefers the *sn*-2 to *sn*-1 acyl chain of phospholipids as an acyl donor [16]. Although *Plaat5*^−/−^ mice have large reductions in various NAEs in the testis, substantial amounts of NAEs are still present. These remaining NAEs are at least partially synthesized by cPLA_2_ε, as a 23% decrease in NAEs is also observed in the testis of *Pla2g4e*^−/−^ mice [71]. In contrast, the levels of NAPEs show only a decreasing trend in *Plaat5*^−/−^ testis. This is probably because a substantial portion of NAPEs may be supplied from other sources through the bloodstream [70,72,73,74]; NAPEs may be rapidly metabolized to downstream NAEs or related lipids; or there may be additional NAPE-biosynthetic enzyme(s) other than PLAAT5, PLAAT1, and cPLA_2_ε in the testis. In support of the second possibility, other NAE-related lipids downstream of NAPEs, such as GP-NAEs and free fatty acids, are also decreased in *Plaat5*^−/−^ mice.

**Figure 6:**
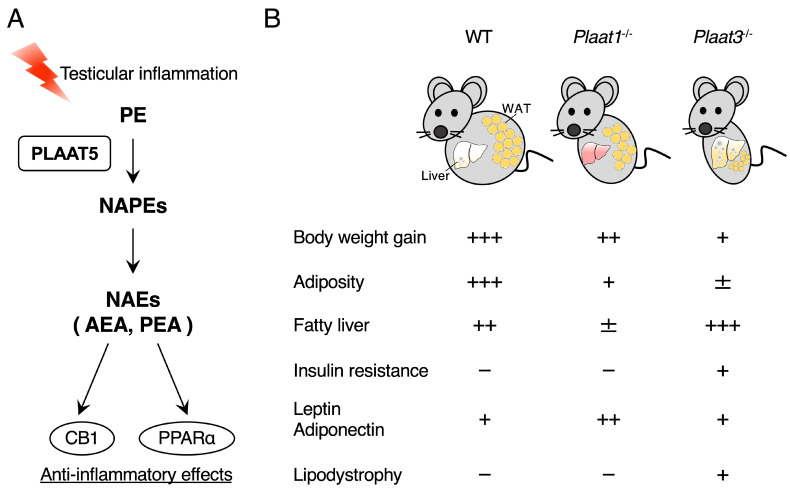
Physiological roles of the PLAAT proteins. (**A**) In the testicular inflammation model, NAEs biosynthesized by PLAAT5 exert anti-inflammatory effects through CB1 or PPARα. (**B**) The phenotypic comparison of WT, *Plaat1*^−/−^ and *Plaat3*^−/−^ mice following HFD feeding. *Plaat1*^−/−^ and *Plaat3*^−/−^ mice show resistance to HFD-induced obesity with distinct effects on the liver and WAT. The symbols +, −, and ± denote positive, negative, and weak phenotypes, respectively, and the number of these symbols indicates the severity of the phenotypes. For details, please see the text.

PEA/NAE-16:0 and AEA/NAE-20:4, known as anti-inflammatory NAEs (Figure 1) [1,2,7,75], are significantly decreased in *Plaat5*^−/−^ mice. Consistently, *Plaat5*^−/−^ mice are more susceptible to testicular inflammation induced by cadmium chloride (CdCl_2_), an environmental toxin [76,77], than WT mice. In this testicular inflammation model, the expression of inflammatory genes, such as *Il6*, *Tnf*, and *Nos2*, is significantly higher in the testis of *Plaat5*^−/−^ mice than in that of WT mice. The administration of exogenous PEA or AEA attenuates the inflammatory gene expression, while cotreatment with antagonists against the PEA receptor PPARα or the AEA receptor CB1 abolishes the anti-inflammatory effects of exogenous PEA and AEA. These results suggest that PLAAT5 exerts anti-inflammatory effects through the production of testicular NAEs, especially PEA and AEA. Since CdCl_2_ treatment does not affect PLAAT5 expression or NAE levels in the testis, PLAAT5 is involved in the maintenance of basal levels of NAE-related lipids in this tissue (Figure 6A).

The fact that PLAAT5 is specifically and constitutively expressed in the testis but not in any other tissues implies its potential involvement in testis-related functions, such as spermatogenesis and fertility. The testis is one of the organs that are rich in DHA, an essential ω-3 polyunsaturated fatty acid [78]. Knockout mice deficient in enzymes involved in DHA metabolism, such as group III secreted PLA_2_ [79], lysophosphatidic acid (lysoPA) acyltransferase 3 [80], and acyl-CoA synthetase 6 [81], display severe male infertility with abnormal sperm morphology, suggesting that lipids containing DHA are required for proper spermatogenic processes. Despite significant decreases in DHA-containing NAE (DHEA) and GP-NAE (GP-DHEA) in the testis, *Plaat5*^−/−^ mice are fertile and have normal testicular weight and sperm numbers. Thus, it is unclear whether PLAAT5, as well as its metabolites of DHA-containing DHEA and GP-DHEA, have some testis-related functions, which warrants further investigations.

### 3.3. Deficiency of PLAAT1 or PLAAT3 Ameliorates High-Fat Diet-Induced Obesity

Unlike PLAAT5 as a testis-specific *N*-acyltransferase, PLAAT1 is expressed in skeletal muscle, the heart, testis, and liver and displays both *N-*/*O*-acyltransferase and PLA_1_/A_2_ activities [58,82], while PLAAT3/PLA2G16 is highly expressed in white adipose tissue (WAT) and displays PLA_1_/A_2_ activity predominantly [56,83]. Both *Plaat1*^−/−^ and PLAAT3-deficient (*Plaat3*^−/−^) mice are born at the expected Mendelian frequency, are fertile, and are viable [84]. Under standard diet feeding, the body weight of *Plaat1*^−/−^ mice is comparable to that of WT mice, whereas *Plaat3*^−/−^ mice show a lean phenotype with markedly less fat mass. A high-fat diet (HFD) significantly increases body weight in WT mice, whereas the body weight gain is moderate in *Plaat1*^−/−^ mice and negligible in *Plaat3*^−/−^ mice. WAT weight shows a similar trend, with a marked increase in WT mice, a moderate increase in *Plaat1*^−/−^ mice, and a negligible increase in *Plaat3*^−/−^ mice. Striking differences are observed in the liver, where HFD feeding markedly increases liver weight in WT mice and even more so in *Plaat3*^−/−^ mice, but not in *Plaat1*^−/−^ mice that are resistant to an HFD-induced fatty liver. In addition, *Plaat3*^−/−^ mice, but not *Plaat1*^−/−^ mice, develop insulin resistance even under standard diet conditions. The phenotypes observed in *Plaat3*^−/−^ mice, i.e., low adiposity with the ectopic accumulation of hepatic fat and increased insulin resistance, are representative of the typical features of lipodystrophy [85]. A similar lipodystrophic phenotype, with the marked impairment of adipocyte differentiation and increased lipolysis in WAT in *Plaat3*^−/−^ mice, was also demonstrated by Sul’s group [83]. Consistent with these observations, recent whole-exome and whole-genome sequencing analyses have identified *PLAAT3* to be a causative gene for human lipodystrophy syndrome [86]. These results indicate that although the deficiency of PLAAT1 and PLAAT3 confers resistance to HFD-induced obesity, the underlying mechanisms are different (Figure 6B).

Lipidomic analysis of the liver from *Plaat1*^−/−^ mice reveals that phospholipids, including PC, PE, PS, and phosphatidylinositol (PI), tend to increase, while lysophospholipids, such as lysoPC, lysoPS, lysoPI, and lysoPA, tend to decrease [84]. Similar changes in the phospholipid profile, accompanied by protection against HFD-induced obesity and hepatic steatosis, have also been observed in mice deficient in the Ca^2+^-independent PLA_2_ group VIA (iPLA_2_β, also known as PLA2G6 or PNPLA9) [87] or group VIB (iPLA_2_g or PNPLA8) [88,89], suggesting the importance of the proper phospholipid/lysophospholipid ratio for hepatic homeostasis [21]. As for *Plaat3*^−/−^ mice, Sul’s group proposed that the reduced adiposity might rely on a decrease in PGE_2_, a PLA_2_-driven arachidonic acid metabolite, and thereby the reduced signaling of its receptor EP3 in WAT [83]; however, the causal relationship between the reduced PGE_2_-EP3 signaling and lipodystrophy is unclear since none of the knockout mice for PGE_2_ synthases or receptors displayed a similar phenotype. Nevertheless, since NAPE and NAE levels were unchanged in the skeletal muscle, heart, testis, and liver of *Plaat1*^−/−^ mice [84] or WAT of *Plaat3*^−/−^ mice, neither PLAAT1 nor 3 appears to function as an *N*-acyltransferase, but rather they act mainly as PLA_1_/A_2_, at least in these tissues. These results suggest that the metabolic phenotypes observed in *Plaat1*^−/−^ and *Plaat3*^−/−^ mice are unrelated to NAPEs and NAEs but are instead caused by an altered phospholipid/lysophospholipid ratio or other mechanisms, such as the dysfunction of organelle homeostasis, as described below.

### 3.4. Roles of PLAAT Proteins in Organellar Membrane Degradation

Compelling evidence has recently indicated that PLAAT proteins play a critical role in maintaining organelle homeostasis in a manner dependent on their enzyme activity [90,91]. The expression of PLAAT proteins, except for PLAAT5, in mammalian cells exerts various effects on the organelle structure, including peroxisomal degradation and mitochondrial fragmentation (Table 2) [13,92,93,94,95]. These functions of PLAAT proteins are associated with the bulk degradation of various organelles, such as the endoplasmic reticulum, lysosomes, mitochondria, and peroxisomes, during the maturation of the eye lens [96], thereby allowing optimal transparency. The eye lens of *Plaat3*^−/−^ mice and *plaat1*^−/−^ zebrafish fails to degrade organelles and eventually develops cataracts. PLAAT3 is recruited to the damaged organellar membranes and disrupts the whole organelle by its PLA_1_/A_2_ activity. Autophagy is known to play a critical role in maintaining cellular homeostasis by degrading not only proteins and lipids but also organelles, and its dysfunction has been implicated in various diseases, including obesity and fatty livers [97,98,99], implying the importance of proper organelle quality control. Therefore, it is of great interest to clarify whether the organelle degradation caused by PLAAT proteins is an alternative method of organelle quality control, in general, and whether this mechanism is responsible for the metabolic phenotypes of PLAAT-deficient mice, such as resistance to HFD-induced fatty livers in *Plaat1*^−/−^ mice and the WAT atrophy and insulin resistance in *Plaat3*^−/−^ mice. In this context, the quality control of organelles by PLAAT3 may also be associated with virus infection, where the inactivation of PLAAT3 in mammalian cells results in resistance to picornavirus infection [100,101]. Indeed, while WT mice succumb to infection with picornaviruses (coxsackievirus A4 or coxsackievirus A10) and show symptoms such as paralysis by 1 week, this contrasts with *Plaat3*^−/−^ mice, which can survive without signs of illness. Mechanistically, PLAAT3-mediated endosomal membrane hydrolysis and pore formation may allow the entry of viral genomes into the cytosol.

### 3.5. Other PLAATs

PLAAT2 and 4 exist in primates but not in rodents, and therefore, it is difficult to study their physiological roles using knockout strategies. With the aid of a chemically inducible dimerization system targeting the protein of interest to the mitochondria, it has been demonstrated that PLAAT2 and 4 are capable of degrading organelles, as observed for PLAAT1 and 3 (Table 2) [94]. While the analysis of PLAAT2 has not progressed much, PLAAT4 has been associated with retinoid-regulated cell growth [102,103,104], virus and parasite infection [105,106,107], and keratinocyte differentiation [108,109]. Although the underlying mechanisms are not yet determined, the enzymatic activity of PLAAT4 is required for these functions, suggesting a key role for the PLAAT4-driven production of certain lipid metabolites or the degradation of organellar membranes. Further research, including human genomics and lipidomic analyses, will expand our understanding of the biological functions of PLAAT2 and 4 and their involvement in human diseases.

## 4. Concluding Remarks

In this review, we summarize recent advances in some unique biological roles of the PLA_2_ superfamily, mainly focusing on cPLA_2_ε and PLAAT proteins. cPLA_2_ε and PLAAT5 function as *N*-acyltransferases and produce anti-inflammatory NAEs to mitigate pathological conditions, including psoriasis, brain ischemia, and testicular inflammation. Deficiency in PLAAT1 and 3 causes resistance to HFD-induced obesity with distinct effects on the liver and WAT. While these observations have expanded our knowledge of these enzymes, there are still many open questions that need to be addressed. Regarding the biosynthetic pathways and physiological functions of NAEs, (i) lipidomic analyses of *Pla2g4e*^−/−^ and *Plaat5*^−/−^ mice have indicated the presence of additional *N*-acyltransferase(s), which await molecular identification alongside the analysis of enzymatic properties and biological functions. (ii) It remains unclear whether cPLA_2_γ, GDE4, and GDE7 are indeed involved in NAE biosynthesis in vivo, which needs further confirmation using knockout animals. As for organelle degradation by PLAAT proteins, (iii) although they have been shown to translocate to a damaged organelle membrane through their C-terminal hydrophobic domain [100], the mechanisms by which the organelle membrane is damaged remain unclear. (iv) It has yet to be determined whether PLAAT-mediated organelle degradation is related to the phenotypes of *Plaat1*^−/−^ and *Plaat3*^−/−^ mice. Studies to answer these questions will provide further insights into the physiological roles of cPLA_2_ε and PLAAT proteins, as well as their potential as therapeutic targets in various diseases, including psoriasis, brain ischemia, inflammation, and obesity.

## Figures and Tables

**Table 1:** Enzymatic properties of the cPLA_2_ family. The functions of cPLA_2_a and cPLA_2_e have been confirmed by in vivo studies, whereas those of cPLA_2_b, g, d, and z are based on only in vitro studies and need to be confirmed in vivo in future studies. The symbols +, −, and ± denote positive, negative, and faint enzyme activities, respectively, and the number of these symbols indicates the magnitude of the enzyme activity. ND, not detected.

Name Enzyme (Gene)	Enzyme Activity			Ca^2+^ Dependency
	PLA_1_/A_2_	Lysophospholipase	Transacylase	
cPLA_2_α (*PLA2G4A*)	++ (PLA_2_)	±	±	Yes
cPLA_2_β (*PLA2G4B*)	+ (PLA_1_ = PLA_2_)	++	ND	Yes
cPLA_2_γ (*PLA2G4C*)	++ (PLA_2_)	++	++ (*O*-acyltransferase)	No
cPLA_2_δ (*PLA2G4D*)	+ (PLA_1_ > PLA_2_)	+	+ (*O*-acyltransferase)	Yes
cPLA_2_ε (*PLA2G4E*)	±	−	++ (*N*-acyltransferase)	Yes
cPLA_2_ζ (*PLA2G4F*)	++ (PLA_2_)	+	ND	Yes

**Table 2:** Enzymatic properties of the PLAAT family. The symbols +- and ± denote positive, negative, and faint enzyme activities, respectively, and the number of these symbols indicates the magnitude of enzyme activity. ND, not detected.

Name	Synonym	Enzyme Activity			Organelle- Degrading Activity
		PLA_1_/A_2_	*N*-Acyltransferase	*O*-Acyltransferase	
PLAAT1	A-C1, HRASLS1	+	++	++	Yes
PLAAT2	HRASLS2	++	+++	++	Yes
PLAAT3	AdPLA, HRASLS3, H-rev107, PLA2G16	+++	±	+	Yes
PLAAT4	HRASLS4, RARRE3, RIG1, TIG3	++	±	+	Yes
PLAAT5	HRASLS5, iNAT	+	+	+	ND

## Data Availability

The dataset is available on request to the authors.

## Abbreviations

The following abbreviations are used in this manuscript:

ABHD4	α/β-hydrolase domain-containing 4
AD	Alzheimer’s disease
AdPLA2	adipose-specific PLA2
AEA	N-arachidonoylethanolamine
CdCl2	cadmium chloride
cPLA2	cytosolic phospholipase A2
DHA	docosahexaenoic acid
DHEA	N-docosahexaenoylethanolamine
DPEA	N-docosapentaenoylethanolamine
EPA	eicosapentaenoic acid
GDE	glycerophosphodiesterase
GP-NAE	glycerophospho-NAE
HFD	high-fat diet
IMQ	imiquimod
iPLA2	Ca2+-independent phospholipase A2
LRAT	lecithin-retinol acyltransferase
lysoPA	lysophosphatidic acid
NAE	N-acylethanolamine
NAPE	N-acyl-phosphatidylethanolamine
NAPE-PLD	NAPE-hydrolyzing phospholipase D
NAPS	N-acyl-phosphatidylserine
OEA	N-oleoylethanolamine
PA	phosphatidic acid
PC	phosphatidylcholine
PE	phosphatidylethanolamine
PEA	N-palmitoylethanolamine
PI	phosphatidylinositol
PIP2	phosphatidylinositol 4,5-bisphosphate
PLA1	phospholipase A1
PLA2	phospholipase A2
PLAAT	phospholipase A and acyltransferase
PPARα	peroxisome proliferator-activated receptor α
PS	phosphatidylserine
SEA	N-stearoylethanolamine
WAT	white adipose tissue
WT	wild type
